# A Web-Based Data Collection Platform for Multisite Randomized Behavioral Intervention Trials: Development, Key Software Features, and Results of a User Survey

**DOI:** 10.2196/resprot.6768

**Published:** 2017-06-16

**Authors:** Riddhi A Modi, Michael J Mugavero, Rivet K Amico, Jeanne Keruly, Evelyn Byrd Quinlivan, Heidi M Crane, Alfredo Guzman, Anne Zinski, Solange Montue, Katya Roytburd, Anna Church, James H Willig

**Affiliations:** ^1^ University of Alabama at Birmingham Birmingham, AL United States; ^2^ University of Michigan Ann Arbor, MI United States; ^3^ John Hopkins University Baltimore, MD United States; ^4^ University of North Carolina at Chapel Hill Chapel Hill, NC United States; ^5^ University of Washington Seattle, WA United States

**Keywords:** iENGAGE, software design, behavioral research, survey, Web application, HIV, user perspective

## Abstract

**Background:**

Meticulous tracking of study data must begin early in the study recruitment phase and must account for regulatory compliance, minimize missing data, and provide high information integrity and/or reduction of errors. In behavioral intervention trials, participants typically complete several study procedures at different time points. Among HIV-infected patients, behavioral interventions can favorably affect health outcomes. In order to empower newly diagnosed HIV positive individuals to learn skills to enhance retention in HIV care, we developed the behavioral health intervention Integrating ENGagement and Adherence Goals upon Entry (iENGAGE) funded by the National Institute of Allergy and Infectious Diseases (NIAID), where we deployed an in-clinic behavioral health intervention in 4 urban HIV outpatient clinics in the United States. To scale our intervention strategy homogenously across sites, we developed software that would function as a behavioral sciences research platform.

**Objective:**

This manuscript aimed to: (1) describe the design and implementation of a Web-based software application to facilitate deployment of a multisite behavioral science intervention; and (2) report on results of a survey to capture end-user perspectives of the impact of this platform on the conduct of a behavioral intervention trial.

**Methods:**

In order to support the implementation of the NIAID-funded trial iENGAGE, we developed software to deploy a 4-site behavioral intervention for new clinic patients with HIV/AIDS. We integrated the study coordinator into the informatics team to participate in the software development process. Here, we report the key software features and the results of the 25-item survey to evaluate user perspectives on research and intervention activities specific to the iENGAGE trial (N=13).

**Results:**

The key features addressed are study enrollment, participant randomization, real-time data collection, facilitation of longitudinal workflow, reporting, and reusability. We found 100% user agreement (13/13) that participation in the database design and/or testing phase made it easier to understand user roles and responsibilities and recommended participation of research teams in developing databases for future studies. Users acknowledged ease of use, color flags, longitudinal work flow, and data storage in one location as the most useful features of the software platform and issues related to saving participant forms, security restrictions, and worklist layout as least useful features.

**Conclusions:**

The successful development of the iENGAGE behavioral science research platform validated an approach of early and continuous involvement of the study team in design development. In addition, we recommend post-hoc collection of data from users as this led to important insights on how to enhance future software and inform standard clinical practices.

**Trial Registration:**

Clinicaltrials.gov NCT01900236; (https://clinicaltrials.gov/ct2/show/NCT01900236 (Archived by WebCite at http://www.webcitation.org/6qAa8ld7v)

## Introduction

In multisite behavioral intervention trials, participant enrollment, randomization, data collection, security, storage and access, and intervention fidelity are all components critical to the success of a study [1,2]. However, recruitment activities for multisite trials are challenging [3]. Meticulous tracking of study data must begin early in the study recruitment phase and must account for regulatory compliance, minimize missing data, and provide high information integrity and reduction of errors [4,5]. Central study coordinators are typically tasked with tracking and documenting participant activities [3,4]. Electronic data capture is a necessity for multisite randomized clinical trials and is replacing the practice of manual data entry on paper forms [4]. There are several electronic data capture systems available for the conduct of randomized clinical trials, and while there are some examples of behavioral sciences research being supported by software [2], this practice is much less common than in the clinical trials space. Like most randomized clinical trials, in behavioral intervention trials, participants typically complete several study procedures at different time points. Prior studies indicate that a single coordinator may simultaneously work on more than one study and customized study software solutions that allow accurate tracking and documentation of encounters are needed [4]. With limited literature available on multisite behavioral intervention trials supported by custom software, there is little opportunity for such investigators to learn how this technology could facilitate the accomplishment of their research goals.

Among HIV-infected patients, behavioral interventions can favorably affect health outcomes. In particular, the events of the first year after diagnosis are critical to achieve favorable long-term health outcomes. While patients adjust to a new diagnosis, they must develop behavioral skills to simultaneously fully engage and remain in HIV care longitudinally [6]. Adherence to HIV primary care appointments and antiretroviral therapy (ART) during the first year is associated with achieving clinical care milestones like HIV viral load (VL) suppression [7], a marker of effective HIV therapy, and decreased mortality [8,9]. In order to empower newly diagnosed individuals living with HIV to learn skills to enhance retention in HIV care, we developed the behavioral health intervention Integrating ENGagement and Adherence Goals upon Entry (iENGAGE) funded by the National Institute of Allergy and Infectious Diseases (NIAID). In this trial, we deployed an in-clinic behavioral health intervention in 4 urban HIV outpatient clinic sites in the United States. In order to scale our intervention strategy homogenously across 4 US clinic sites, we to developed software for this study that would function as a behavioral sciences research platform.

We identified a need to develop a Web-based platform to collect and maintain data uniformly and to serve as a resource for interventionists implementing a behavioral science intervention to improve outcomes during the first year of HIV care. In this manuscript, we aimed to (1) describe the design and implementation of a Web-based platform to facilitate the deployment of a multisite behavioral science intervention; and (2) report on the results of a survey to capture end-user perspectives of the impact of this platform on the conduct of a behavioral intervention trial.

## Methods

### Overview iENGAGE

iENGAGE is a National Institutes of Health (NIH)/NIAID-funded (R01 AI 103661) in-clinic behavioral intervention trial designed to evaluate intervention efficacy relative to standard of care for improving treatment outcomes among patients initiating HIV medical care. This multisite randomized controlled trial (RCT) implemented a comprehensive intervention arm that combines 2 previously tested approaches—Centers of Disease Control (CDC) Retention in Care (RIC) [4] and Participating and Communicating Together (PACT) [5] to support the establishment of early behaviors that help patients to arrive at scheduled medical appointments and learn to take ART medications as prescribed.

### Setting

The implementation sites were the University of Alabama at Birmingham (UAB), the University of North Carolina at Chapel Hill (UNC), Johns Hopkins University (JHU) in Baltimore, and the University of Washington in Seattle (UW). The institutional review boards (IRB) at each participating site approved this protocol.

### Study Team

The research team consisted of a principal investigator (a physician with more than 20 years of research experience), a study coordinator, 2 research assistants, and 2 interventionists. The software development team consisted of a senior designer (a physician with more than 20 years of research experience), a network and security expert, 2 programmers, and a data analyst.

The UAB/overall study coordinator was integrated into the informatics team and became a regular participant in software development meetings, also serving as a liaison to the principal investigator and the site coordinators at UNC, UW, and JHU. The overall study coordinator was empowered by the principal investigator to have final approval of changes to the software to facilitate study workflow.

### Software Development

Software development took place within the UAB Research and Informatics Service Center (RISC). We used a user-centered design approach and integrated the overall and UAB iENGAGE study coordinator and coordinator responsible for study implementation across the 3 other study sites into the software development process. Initial meetings focused on cementing the understanding of the study protocol and means to conduct the intervention evaluation uniformly across the 4 sites.

We developed iENGAGE as a Web-based application using C# and .NET, which use a 128-bit encryption Secure Sockets Layer (SSL) certificate to protect any data transmitted to or from the application and all user passwords were hashed into the user table. This Web-based application is accessible from any computer and does not require local installation. Each user across the 4 US HIV clinics, can access the software with their distinct user name and password, and embark on real-time data capture at the point of care (ie, exam rooms before or after physician visit) via the Internet-connected device of the user's choice (eg, laptop, tablet, desktop, etc). Keeping participant confidentiality in mind, we made every effort to include minimal protected health information (PHI) and securely host data in UAB Health System Servers using the Oracle Relational Database Management System (RDMS). Apart from being an informational data repository, this Web application actively informs workflow by firing alerts at scheduled times for study activities for enrolled patients [10,11]. Our design had to accommodate screening, enrollment, and intervention activities during total study duration.

We defined the key features for the software after extensive and repeated discussion with stakeholders at various times. The first series of discussions began before grant submission, when we met with the principal investigator, participated in the initial study design, and collaboratively conceptualized the overall features the software would need to incorporate to support the planned intervention. After the grant was funded, regular meetings with the study team took place. During these meetings, the research team finalized the proposed study workflow, while the design of the software needed to support the intended data collection was prototyped concurrently.

The following is a list of key features needed to support multisite behavioral sciences research that arose from these meetings: (1) study screening and enrollment, (2) participant randomization, (3) real-time data collection by study personnel and participants, (4) facilitation of longitudinal workflow, (5) reporting, and (6) reusability.

#### Study Screening and Enrollment

**Figure 1:**
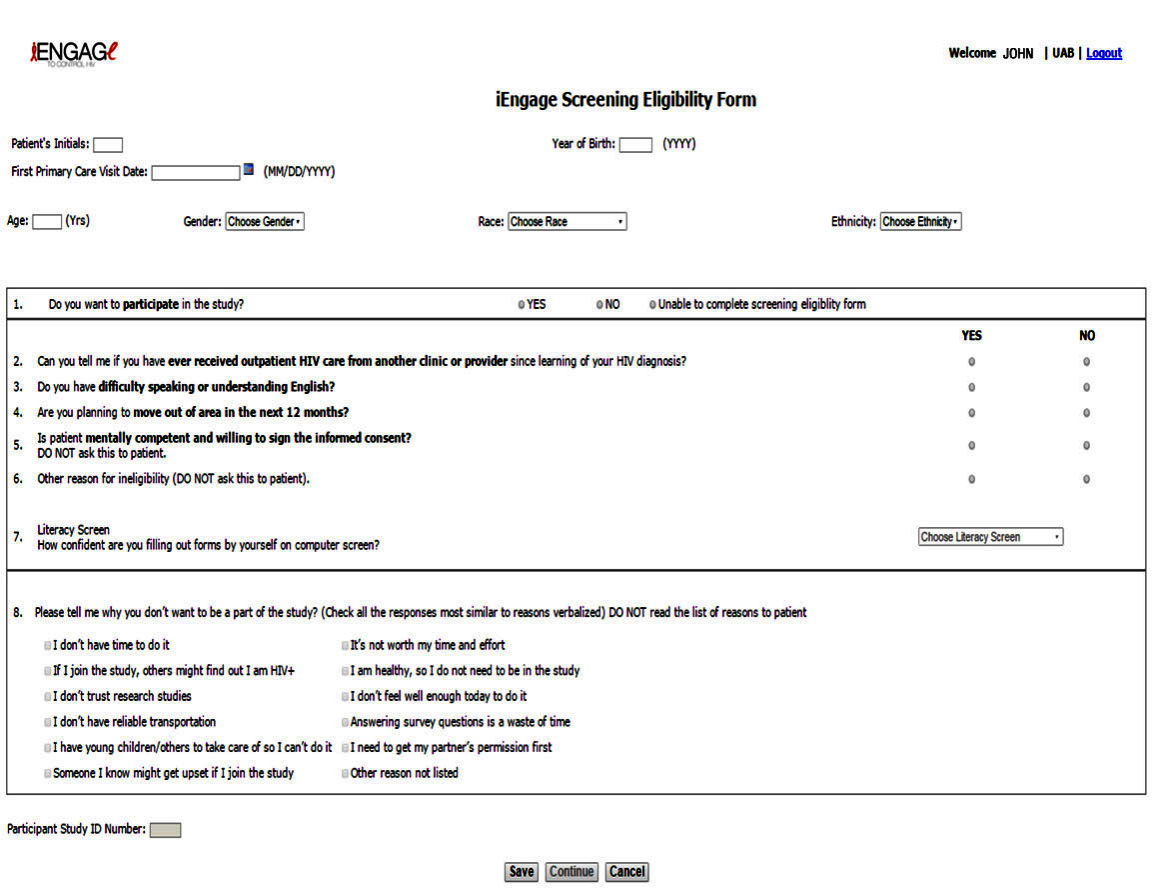
Patient registration.

Upon initiating the enrollment of a potential study participant under the “Registration” tab, users accessed a “screening form” specific to the iENGAGE study that included questions that comprised the study inclusion and exclusion criteria (Figure 1). Once the screening form was completed and saved, the software would inform research staff if the participant was eligible or ineligible to participate in the iENGAGE study. Upon successful screening, a study-specific identifier was automatically assigned to each participant (Figure 1). This centralized method of data collection on recruitment activities ensured consistency across study sites.

#### Participant Randomization

In order to randomize participants equally to study arms across all sites, randomization took place centrally. We used a variant of the permuted block randomization process to complete randomization to a specific study arm (intervention or control) in a 1:1 ratio [12]. First, we used third party statistical software (SAS) to generate a list of random numbers in blocks of 2, 4, or 6 that were loaded into a randomization table in our software. As each participant was enrolled in the study, the next (externally) generated number in the random sequence would come up and, depending on the value, the participant was assigned to the intervention or control arm of the trial. This approach of electronically pre-loading a random sequence of numbers generated by the study team in a statistical software solution of their choice provided our researchers greater facility in regards to our participant randomization process, while at the same time eschewing the need to create code to randomly assign study participants to an arm of the study. This solution allowed for subsequent reusability and customization to varying study sizes.

#### Real-Time Data Collection

The need for real-time, in-clinic capture of information was an important part of functionality. This impacted design and the user experience, necessitating an interface optimized for tablets and continuous Internet connection. This allowed study personnel mobility throughout the clinic and supported interaction at the point of care.

For this behavioral intervention trial, the research team created specific forms for data capture that supported the intended study design and subsequent analyses for inclusion in the software. These study forms included close-ended (eg, single answer “Yes”/“No” or multiple choice questions) and open-ended questions (ie, “Other, please specify”) supported via free text fields within our software. These free text fields supported the capture of unplanned and/or unexpected data for subsequent review by our investigators.

#### Facilitation of Longitudinal Workflow

It was imperative that researchers across multiple sites followed a single study protocol longitudinally. By understanding the study protocol and its timeline, we were able to design forms triggered by the passage of pre-specified time intervals from enrollment that would appear in a study coordinator's worklist automatically. Thus, study coordinators did not have to individually track when participants were due for subsequent interventions and/or contacts (eg, final assessment 12 months after enrollment). In addition, a flag that changed colors from green to yellow to red when a form was overdue accompanied each new form. These functions facilitated both patient tracking throughout the study and contributed to the timeliness of data collection.

#### Reporting

The software was able to generate pre-specified data reports upon request that would allow evaluation of enrollment milestones from each study location as well as indicating for each participant their location on the study protocol timeline. We pulled data on a recurring monthly basis to conduct audits on the study status at each of the 4 sites. These reports facilitated both site-specific and central monitoring of fidelity and adherence to study protocol, by indicating cases where the study protocol was not being met and triggering appropriate and timely action. In case of additional ad hoc data requests, our study coordinator submitted a formal query form detailing requested data elements to the informatics team at UAB. Such ad hoc requests could potentially be added as pre-specified reports if needed.

Data was provided in Microsoft (MS) Excel, MS Access, and comma-separated values (CSV) files a format that could be imported into various available statistical analysis software packages (eg, SAS, STATA, etc).

#### Reusability

A key principle of our design was reusability. As our group supports multiple ongoing behavioral science studies, we strove to build functionality that would support this line of research. Thus, we focused our design on features that would provide flexibility to support future study protocols, such as customizable worklists, reporting, and participant randomization functions.

This software has positively impacted our behavioral science researchers. By utilizing Web-accessible software to support behavioral science, research stands to greatly benefit participants and investigators as it allows for data capture outside of clinics in patient’s homes and community settings and supports a branch of research that has had limited access to such tools in contrast to clinical trials. This software also benefits participants by empowering those that, due to disability or other limitation, cannot participate in research in traditional clinical settings. In fact, we have used this database for other ongoing behavioral intervention trials like Birmingham Access to Care (BA2C) and will be using it in other projects for community testing and linkage to care for HIV, hepatitis C, and sexually transmitted infections in 2017.

### Testing

We implemented database testing initially at UAB during which we performed a variety of study activities using the software.

During the initial round of testing all UAB (ie, central site) users (N=5) completed a round of one-on-one testing of the software and reported findings to the development team. During testing, each prospective user added fictional participants (equal to or greater than 1) to the database and completed the forms associated with the study with the goal of following study workflow from enrollment to completion. The research team users provided feedback directly to the software development team. We reviewed proposed changes with the study coordinator, and once approved, made these changes to the initial software prototype. A second round of testing at UAB involved a user group simultaneously following a fictional participant from enrollment to study conclusion. Representatives from the software development team attended this session and collated feedback for subsequent review with the study coordinator. From this second round, we added additional iterative changes to the software and it was deemed ready for external testing.

External testing was divided into 2 rounds; a one-on-one round followed by a group session by all external users (N=8). We collated feedback from each round and reviewed with the study coordinator who had final approval of proposed changes to the software.

Study recruitment started at UAB and served as a 1-month pilot period that allowed further software testing during real-world workflow conditions. Before we started the study recruitment procedures at UAB, all major changes were completed. At this point, we can say that there were no major changes made to the database. Before launch, each participating site took part in an online webinar to provide training on new functionality. We collected feedback continuously from each site by the central study coordinator who communicated with the development team in regular meetings, which led to further refinements in software functionality as appropriate. There were some minor front-end changes to the user interface that included some fields for data collection per the request of the additional 3 participating sites once they had started recruiting.

### Additional Features, Roles, and Functionality

This Web application supported multiple simultaneous users. We defined several user types and their corresponding access depending on their study roles (eg, research assistants, study coordinators, and interventionists). We assigned users a discrete user name and password to login. Once logged in, the home screen displayed tabs to register and search for enrolled participants. The “Registration” tab allowed users to complete the screening activity for potential participants that determined if a participant was eligible, or ineligible, for the iENGAGE study, assigning a study identification (ID) for the former. The “Search” tab allowed users to search for already registered participants and look at all the completed forms. This allowed for a quick review of a participant’s study-related history.

**Figure 2:**
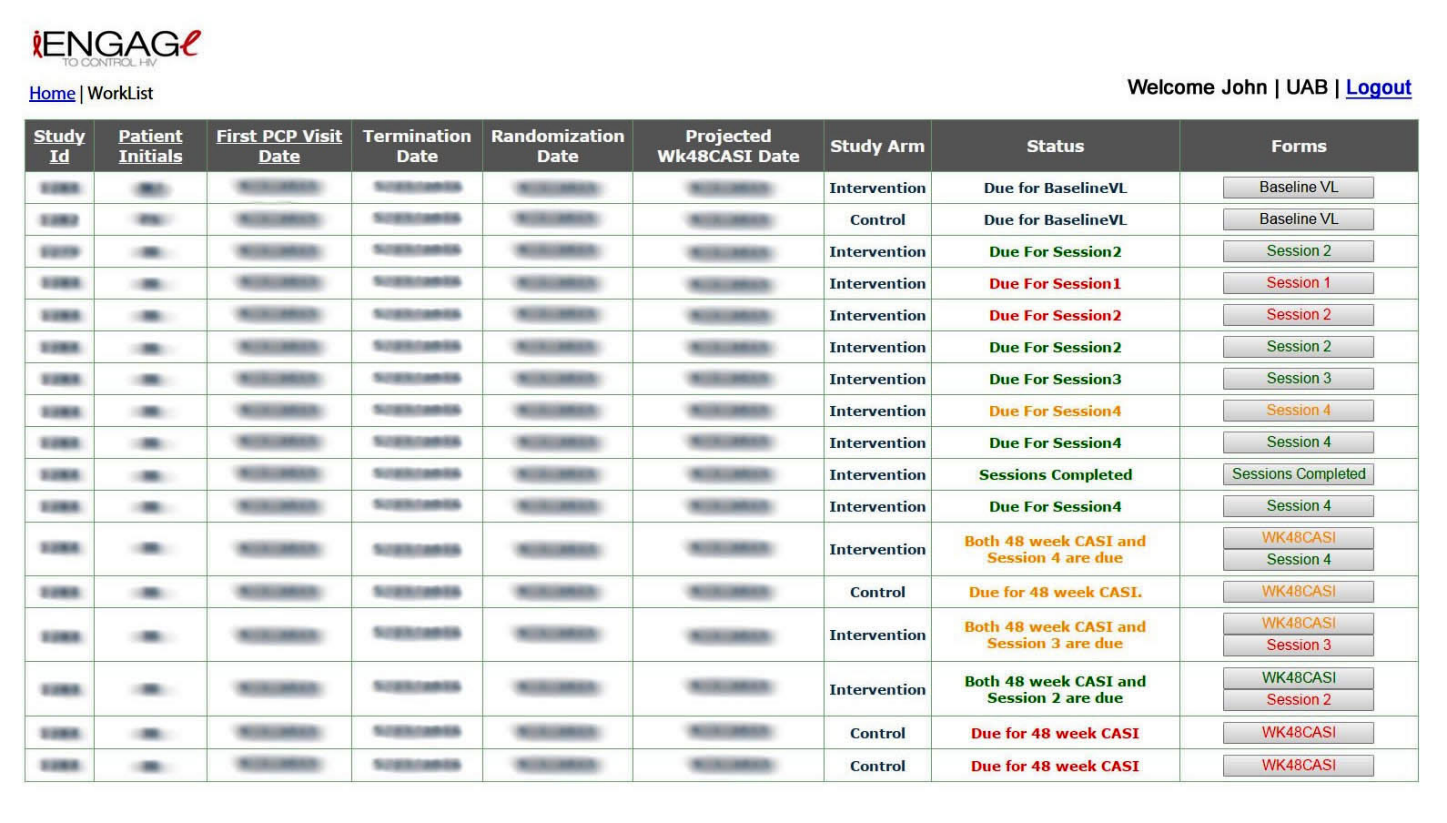
Worklist.

We designed the worklist to provide a clear and easily accessible task list. It differed slightly for team members depending on their roles in the study (eg, research assistants, study coordinators, and interventionists). For research assistants and study coordinators, it displayed a summary of participants screened, their status on the study at that point, along with upcoming study activities for each participant (Figure 2). For the interventionists, the worklist presented pending tasks among intervention arm participants shortly before they were due, minimizing the chance of intervention activity deviations to maximize fidelity in delivery.

### Data Quality and Security

We introduced features in our design to enhance data quality wherever possible and minimize subsequent data cleaning on the back-end. For example, we pre-populated fields with pre-existing data as much as possible to avoid duplication of effort and prevent transcription errors (eg, participant ID and enrollment date). Field controls (date or response ranges) and mandatory fields and/or form completion where indicated by the study team were added. For instance, intervention sessions had mandatory forms like a risk screener and a face-to-face appointments form; users could not move to the next session unless they completed all required forms for the prior session. Once the forms were saved on the system, users were not allowed to make edits. Administrative rights were required to make any changes to the database and all such changes were made centrally. This Web-based application was designed in a way that each user had a unique username and password to login to the website. Therefore, multiple users could access the software simultaneously but not using the same account. No user could override recorded data. In case a documentation error was recognized, our policy was to communicate and clear the issue with the study coordinator and the principal investigator. Thus, only requests approved by the principal investigator and clearly representing a data entry error were changed while data collection was ongoing.

Data are stored and maintained on secure UAB servers where other health system patient data are housed. Data were backed up daily and security updates were made in a timely manner regularly to ensure appropriate protections of the database. Data queries privileges were granted only to a RISC data analyst who was accessible to investigators.

### Survey Development and Data Collection

We adopted a user survey from the Web quality instrument published by Aladwani et al to design the iENGAGE research database Web application survey [13]. This 25-item instrument was structured to evaluate user perspectives on research and intervention activities specific to the iENGAGE trial and overall performance of the iENGAGE platform. There were several questions that all study team members responded to and few of which focused solely on research and intervention activities, which were restricted, based on user roles on the study (ie, research staff versus interventionists).

The initial set of questions focused on appearance (ie, color and fonts), adequacy (ie, always up and available with search and navigate options), specific features (ie, tracking study activities and color code schemes), and data collection procedures (ie, accuracy, patient registration, enrollment, and randomization process, up to date participant information, and data organization and security). Another set of questions asked users if it was meaningful to participate and provide feedback in developing this application. We assessed if pilot testing made it easier to understand user responsibilities and if users would recommend involving research team members in designing Web-based applications in future studies. These items were measured using a 7-point Likert scale from 1 (strongly disagree), 2 (disagree), 3 (disagree somewhat), 4 (undecided), 5 (agree somewhat), 6 (agree), to 7 (strongly agree). We assessed general overall experience and satisfaction using a scale ranging from very negative (0%) to very positive (100%). A set of open-ended questions asked users to list the most and least useful feature of the Web application and users shared their opinions on whether there was something that they would like to change or add. We asked users to provide their suggestions and/or recommendations for future improvements. In addition, we asked users with previous experience of using research software applications to compare iENGAGE to those previously utilized. We implemented this paper-based survey to all software users’ at all 4 study sites. A Portable Document Format (PDF) of the survey was sent to all users via email. Users either completed this survey by editing the PDF copy or manually completed a printed copy. All surveys were returned via email to the UAB study coordinator. Survey responses were kept anonymous beyond the receiving team member (UAB study coordinator). All surveys returned to UAB were manually entered, merged, and verified using an Excel spreadsheet which was then imported in SAS for analysis.

### Quantitative Analysis

We calculated descriptive statistics to present percentages of user agreement for the close-ended questions. We calculated agreement of user survey responses where “agreement” is defined as a composite measure of strongly agree, agree, and agree somewhat.

For open-ended questions, we consolidated the feedback and described them in the results.

## Results

Overall, 372 participants were enrolled and randomized across sites using this Web-based platform in the iENGAGE behavioral intervention trial.

Overall, 13 users completed the survey. Of those, 46% (6/13) were research activity users, 38%, (5/13) were intervention activity users, and 15% (2/13) were using both. We found approximately 90% (≈12/13) overall agreement on the appearance and functionality of the Web application survey (Table 1). There was 100% (13/13) agreement in user responses on ease of use of this database for completing participant registration, enrollment activities, randomization process, and tracking intervention sessions using color code flags (Table 1). All users agreed that the database was accurate in maintaining longitudinal workflow accurately (tracking study activities) and in the organization of participant information (Table 1). Nearly 85% (11/13) of the users agreed that it was easy to track baseline or final study assessments with color code flags (Table 1). All users rated their overall experience, and satisfaction was above 80% on a scale of 0% to 100%, where 0% was negative, 50% was neither negative nor positive, and 100% was positive. There was 100% (13/13) user agreement that participation in the database designing and/or testing phase made it easier to understand user roles/responsibilities and recommended participation of the research team in developing a database for future studies.

In reviewing the responses to the open-ended questions, when users where asked about the most useful features of the application, database users across sites acknowledged ease of use, color flags, longitudinal work flow, and data storage in one location as the most useful features. Users highlighted the registration and randomization processes as user-friendly. Checking eligibility and completing an upcoming task within the assigned study window indicated by the color flags were described as a useful functionality. Users also found maintaining longitudinal workflow in accordance with study protocol and its timeline a useful feature.

Users described some issues saving participant forms, security restrictions, and worklist layout as the least useful features on the database. Some users mentioned that there was nothing least useful on the database. Worklist layout or the order in which participants appear on the worklist and the way ad hoc forms were created as some features that users would like to change in the database to enhance functionality. Users would like to add features like edit options, reminder call tracking, and linkage of the database to the calendar within the software. In addition, one suggestion was to add a feature to capture participant clinic appointment information within the database.

## Discussion

### Principal Findings

We successfully designed a Web-based platform specifically for a multisite behavioral intervention trial to consistently capture patient participant data and maximize fidelity in intervention delivery. There is little evidence available on measuring quality constructs of Web-based applications [13], and to our knowledge, there is scant literature evaluating software applications developed to support behavioral sciences research. By conducting a survey to capture end-user perspectives and reporting on the impact of our behavioral science research platform on the conduct of this type of research, we provided information on the value and impact of features for end-users. Survey results underscored ease of use, color flags, and longitudinal workflow (tracking study activities) as effective features of the database. In the survey, after the experience of using the software in the conduct of the study, our users suggested ideas to enhance acceptance and study functionality. We believe the capture of such information iteratively with the software’s utilization has provided important insights that we have used to strengthen the reusability of our behavioral science research platform software and encourage other developers to connect with their users in a similar fashion.

**Table 1:** Survey agreement for close-ended questions on the Integrating ENGagement and Adherence Goals upon Entry (iENGAGE) Web application survey (N=13).

Question		Total, n (%)	Agreement (%)				Undecided (%)
			Overall	Strongly agree	Agree	Somewhat agree	
Appearance								
	Always up and available	13 (100)	100	62	31	8	
	Fonts were properly used	13 (100)	92	46	46		8
	Colors were properly used	13 (100)	92	46	46		8
Functionality								
	Easy to track study activities (baseline, final assessment) with color code schemes added^a^	8 (62)	88	38	38	13	13
	Easy to track intervention sessions with color code schemes added^b^	7 (54)	100	71	29		
	Easy to search participants	13 (100)	92	23	46	23	8
	Easy to navigate through	13 (100)	100	38	54	8	
	Easy to complete patient registration^a^	7 (54)	100	43	43	14	
	Easy to complete enrollment activities (enrollment form, CASI^c^)^a^	8 (62)	100	50	50		
	Easy to complete randomization process^a^	8 (62)	100	50	38	13	
	Accurately tracked participant’s study activities	13 (100)	100	31	69		
	Always up to date with participant information	12 (92)	92	33	50	8	8
	Facilitated organization of participant information	13 (100)	100	31	54	15	
	Ensured security of participant information	13 (100)	92	46	38	8	8
	It was meaningful to participate and provide feedback in the development process of this application as a whole	12 (92)	92	58	33		8
	Easy to understand user roles/responsibilities	9 (69)	100	56	33	11	
	I will recommend participation of research team in developing a research database application for future studies	12 (92)	100	67	25	8	
Overall experience				≥80	≥90	100		
	Please rate your overall experience	12 (92)	25	58	17		
Overall satisfaction				≥80	≥90	100		
	Please rate your overall satisfaction	12 (92)	33	42	25		

^a^Only research team answered these questions.

^b^Only interventionists answered this question.

^c^CASI: computer-assisted self-interview.

^d^With participation in the design and testing phase.

### Comparison With Prior Work

We believe that integrating research and informatics expertise during the initial database design and final testing phases was an essential step towards successful development of the iENGAGE behavioral research software. Typically, the lead site develops and pilot tests a database, which the collaborating sites have to comply. This could be challenging for the participating sites and interfere with systematic data collection of study activities [14] as factors influencing local use are unknown and may impact implementation. All iENGAGE database users (100% agreement) reported that participation in testing and design phases of the database made it easier to understand user roles/responsibilities on the iENGAGE Web application and recommended participation of the entire research team in developing a Web application for future studies. Our results highlight that close collaboration of research and informatics professionals on a software development project is critical to success and our process offers insights on tangible approaches to achieve this integration.

Behavioral intervention trials are an important part of research, particularly in the management of chronic conditions where, despite the availability of therapies, important gaps in adherence and implementation continue to negatively impact patients. Developing software to support the deployment of behavioral health studies can change the ways in which such trials are conducted to facilitate achievement of research goals [15]. Moreover, tailoring software and access to the specific roles of a research team in conducting a trial (eg, research activities versus intervention delivery) is paramount to usability and satisfaction. The need for software that is customized to study requirements and provides secure multisite simultaneous access to facilitate data collection, and is designed to be integrated into clinical care settings where participant time is limited, will continue to grow, and its adaptability to these settings will be an important factor for success. Collaborative approaches to software development and the utilization of surveys to elucidate user insights especially after completion of the study protocol are an important source of data to improve subsequent software design and should be used routinely. These insights have proved to be very valuable to our team and have been integrated in subsequent enhancements to our behavioral science research platform. In the future, we would explore the possibility of integrating this behavioral research software to patients’ electronic health record (EHR) and allow functionality such as study enrollment directly from the clinical record.

### Limitations

The limitations of this study include a relatively small sample size and a limited geographic area (4 sites) in similar urban settings in large academic medical centers; all factors that may have influenced study results. Survey data were collected anonymously, though the anonymity of responses could potentially have been compromised by the limited number of participants. However, we only asked all users to mention their user role on the survey (not any identifiable information) and merged all surveys to present study results. Social desirability bias is also a potential limitation; however, we note that respondents were forthcoming in offering suggestions to enhance the software including current features that were less than optimal.

### Conclusion

The development of software applications to support behavioral research will be a key component of gaining insight into improving disease management in the age of population health. The successful development of the iENGAGE behavioral science research platform validated the approach of early and continuous involvement of the research study team working side-by-side with informatics designers and programmers in development. In addition, we recommend the post hoc collection of quantitative and qualitative data from the users after deployment as this has led to important insights on how to enhance our software. These approaches have resulted in a flexible toolset that will be able to support multiple behavioral science research studies going forward.

## Abbreviations

ART: antiretroviral therapy
ID: identification
iENGAGE: Integrating ENGagement and Adherence Goals upon Entry
JHU: Johns Hopkins University
MS: Microsoft
NIAID: National Institute of Allergy and Infectious Diseases
PDF: Portable Document Format
RISC: Research and Informatics Center
UAB: Alabama at Birmingham
UNC: University of North Carolina at Chapel Hill Baltimore
UW: University of Washington
VL: viral load

## Appendix

Multimedia Appendix 1 CONSORT EHEALTH Publication form.
